# The SPI-20 and SPI-21 T6SS gene clusters from *Salmonella enterica* subspecies *arizonae* encode effector proteins that display antibacterial activity

**DOI:** 10.3389/fmicb.2026.1770997

**Published:** 2026-03-03

**Authors:** Ayleen Parra-Calisto, Carlos J. Blondel, Carla Vargas-del Río, Esteban Fernández-Castillo, Felipe Reyes-Méndez, Victoria Soriano-Mora, Andrea Avilés, Viviana Toledo, Fernanda Salazar-Salas, Patricio Espinoza-Jara, Fernando A. Amaya, Carlos A. Santiviago, Juan A. Asenjo, David Pezoa

**Affiliations:** 1 Department of Chemical Engineering, Biotechnology and Materials, Centre for Biotechnology and Bioengineering (CeBiB), Universidad de Chile, Santiago, Chile; 2 Institute of Biomedical Sciences, Faculty of Medicine, Universidad Andres Bello, Santiago, Chile; 3 Núcleo de Investigación en One Health, Facultad de Medicina Veterinaria y Agronomía, Universidad de Las Américas, Campus Providencia, Santiago, Chile; 4 Laboratorio de Microbiología, Departamento de Bioquímica y Biología Molecular, Facultad de Ciencias Químicas y Farmacéuticas, Universidad de Chile, Santiago, Chile; 5 Escuela de Medicina, Facultad de Salud, Universidad del Alba, Santiago, Chile

**Keywords:** *Salmonella arizonae*, interbacterial competition, T6SS, effector, immunity proteins

## Abstract

The type VI secretion system (T6SS) is a contact-dependent multiprotein apparatus that contributes to interbacterial competition and pathogenesis in many Gram-negative bacteria. *Salmonella* harbors five T6SS gene clusters within the pathogenicity islands SPI-6, SPI-19, SPI-20, SPI-21, and SPI-22, differentially distributed among serotypes. *Salmonella enterica* subspecies *arizonae* (*S. arizonae*) is most frequently associated with reptiles but, in some circumstances, can cause disease in mammals, including humans. Notably, although it encodes both T6SS_SPI-20_ and T6SS_SPI-21_, no report to date has demonstrated the antibacterial activity of either system. In addition, only two putative effector proteins have been previously predicted, though they have not been experimentally validated. In the present study, we demonstrate that both T6SS_SPI-20_ and T6SS_SPI-21_ contribute to interbacterial competition in *S. arizonae* when grown on McConkey agar plates, suggesting a role for bile in T6SS_SPI-20_ and T6SS_SPI-21_ activity. In addition, through bioinformatic analyses, interbacterial competition assays, and heterologous expression, we further characterize the antibacterial activity of a novel antibacterial E/I module (SARI_02625/SARI_02624, encoded in SPI-21) that, in addition to the previously identified evolved VgrG protein SARI_02603 and VgrG2b homolog SARI_02727, contribute to T6SS-dependent antibacterial competition in *S. arizonae*.

## 1 Introduction

The Type VI Secretion System (T6SS) has emerged as a significant fitness and virulence factor for numerous Gram-negative bacteria [reviewed in Wohlfarth et al. (2025)]. The T6SS is a molecular nanomachine comprised of three primary complexes: a contractile tail, a membrane complex, and a baseplate (Logger et al., 2016; Nazarov et al., 2018; Rapisarda et al., 2019; Yin et al., 2019). The contractile tail includes an internal rigid tube made by the polymerization of a hexameric protein called Hcp, where a needle-shaped VgrG protein trimer is assembled at the tip of the tube. VgrG proteins usually interact with proteins that contain an N-terminal PAAR motif, which serves to sharpen the tip of this structure (Shneider et al., 2013; Brunet et al., 2014; Douzi et al., 2014; Zoued et al., 2014; Brunet et al., 2015b; Renault et al., 2018). The inner rigid structure is covered by a contractile sheath formed by the polymerization of TssB and TssC subunits (Leiman et al., 2009; Basler, 2015; Durand et al., 2015). Contraction of the sheath is responsible for providing the necessary energy for the injection of effector proteins into the target cell. These proteins are confined within the Hcp rigid tube and are either bound to VgrG or associated with PAAR proteins (Silverman et al., 2013). Thus, T6SS effectors are secreted fused to VgrG and/or PAAR proteins (known as evolved or specialized effectors) or associated through non-covalent interaction with some core components (cargo effectors) (Whitney et al., 2014; Diniz and Coulthurst, 2015; Ma et al., 2017; Pissaridou et al., 2018). Most T6SS effectors are bacteria-specific, targeting the peptidoglycan (Srikannathasan et al., 2013; Whitney et al., 2013; Berni et al., 2019; Wood et al., 2019) or the FtsZ protein involved in cell division (Ting et al., 2018), and are encoded in bicistronic units with their cognate immunity proteins (conforming effector/immunity pairs, E/I) to prevent self-intoxication and displacement of sibling cells (Russell et al., 2012). Other T6SS effectors are eukaryote-specific, targeting the actin or microtubule cytoskeleton networks (Durand et al., 2012; Lindgren et al., 2013; Schwarz et al., 2014; Heisler et al., 2015; Sana et al., 2015; Aubert et al., 2016; Jiang et al., 2016; Ray et al., 2017; Dutta et al., 2019; Wood et al., 2019), while others are both bacteria- and eukaryote-specific (also known as trans-kingdom effectors), targeting conserved molecules (e.g., NAD) or macromolecules (e.g., DNA, RNA, and phospholipids), or forming pores in biological membranes (Whitney et al., 2015; Tang et al., 2018; Ahmad et al., 2019).

In *Salmonella*, five different evolutionary lineages of T6SS gene clusters have been identified within pathogenicity islands SPI-6, SPI-19, SPI-20, SPI-21, and SPI-22 (Blondel et al., 2009; Fookes et al., 2011; Bao et al., 2019). In *Salmonella,* only few studies have addressed the role played by the T6SSs in inter-bacterial and eukaryotic relationships, and most of our understanding of the contribution of T6SSs to *Salmonella* pathogenesis comes from studies of T6SS_SPI-6_ in *Salmonella* Typhimurium and T6SS_SPI-19_ in *Salmonella* Dublin (Blondel et al., 2010; Mulder et al., 2012; Pezoa et al., 2013, 2014; Sibinelli-Sousa et al., 2020; Xian et al., 2020; Hespanhol et al., 2022). Regarding the repertoire of effector proteins, 43 T6SS effectors and candidate effectors that target different bacterial molecules and structures such as peptidoglycan, cellular membrane, nucleic acids and bacterial ribosomes have been currently identified in a few serotypes (Blondel et al., 2009, 2023; Russell et al., 2012; Benz et al., 2013; Whitney et al., 2013; Koskiniemi et al., 2014; Sana et al., 2016; Ho et al., 2017; Sibinelli-Sousa et al., 2020; Amaya et al., 2022, 2024; Cobo et al., 2022; Hespanhol et al., 2022; Jurėnas et al., 2022).

*Salmonella enterica* subspecies *arizonae* (*S. arizonae*) is a commensal organism commonly associated with reptiles that harbors both T6SS_SPI-20_ and T6SS_SPI-21_, occasionally causing septicemia and mortality in these hosts (Mahajan et al., 2003). Nevertheless, this subspecies has also been responsible for the manifestation of clinical signs in poultry and in a wide variety of mammals, including immunocompromised humans (Riley et al., 1988; Bella et al., 2011; Lee et al., 2016). Notably, while both T6SS_SPI-6_ and T6SS_SPI-19_ have been linked to antibacterial competition, virulence and host colonization in different *Salmonella* serotypes (Blondel et al., 2010, 2013; Libby et al., 2010; Wang et al., 2011, 2019; Mulder et al., 2012; Pezoa et al., 2013; Koskiniemi et al., 2014; Brunet et al., 2015a; Sana et al., 2016; Schroll et al., 2019; Sibinelli-Sousa et al., 2020; Xian et al., 2020), there is no information on the contribution of T6SS_SPI-20_ and T6SS_SPI-21_ to *S. arizonae* fitness, nor on the repertoire of effector proteins encoded within SPI-20 and SPI-21 T6SS gene clusters.

**Figure 1:**
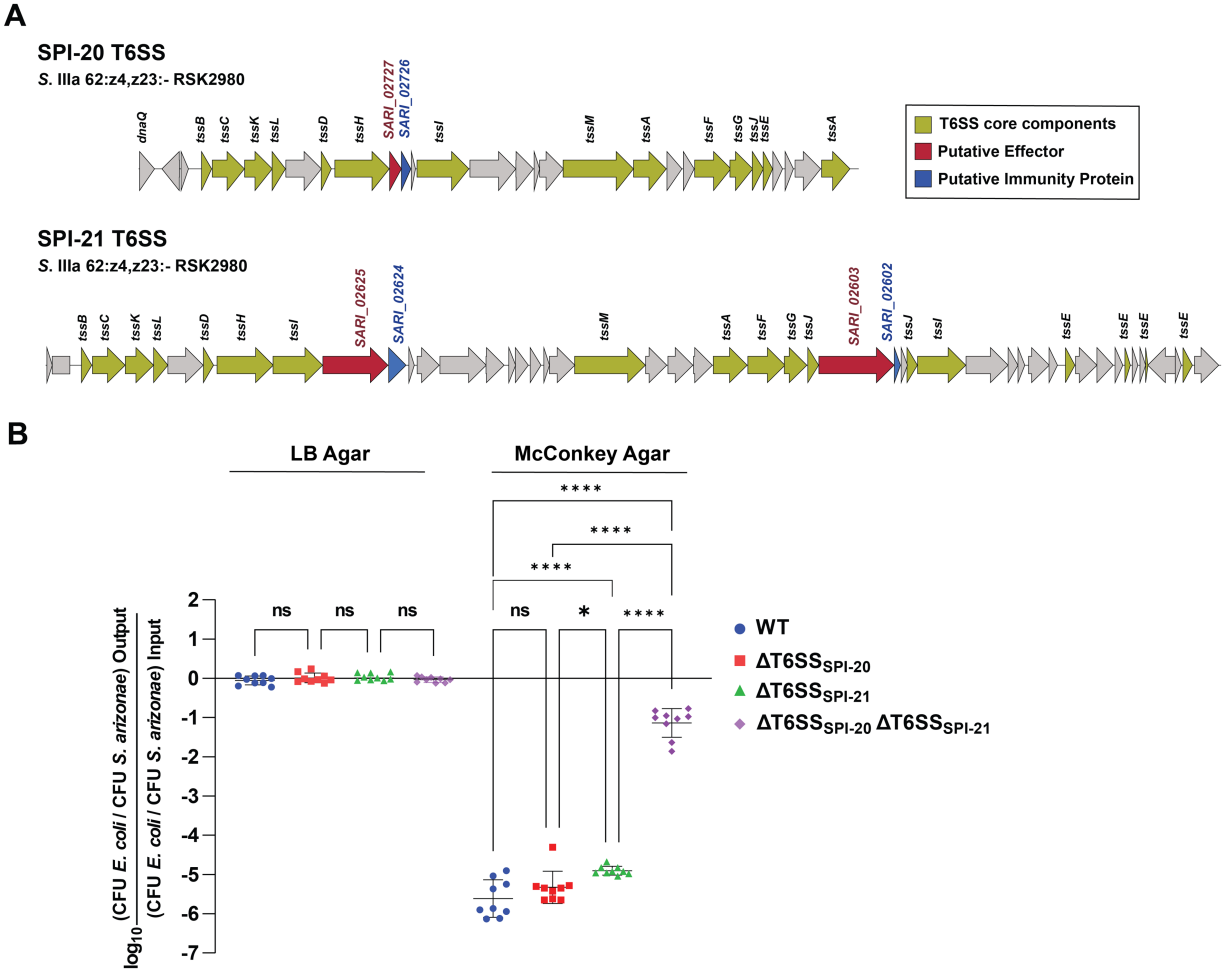
Contribution of T6SS_SPI-20_ and T6SS_SPI-21_ to interbacterial competition by *S. arizonae* RSK2980. **(A)** Scheme of the SPI-20 and SPI-21 T6SS gene clusters of *S. arizonae* RSK2980. **(B)** Interbacterial competition experiments. Wild type and mutant strains ΔT6SS_SPI-20_, ΔT6SS_SPI-21,_ and ΔT6SS_SPI-20_ ΔT6SS_SPI-21_ of *S. arizonae* RSK2980 were mixed at a ratio of 1:1 (attacker/prey) with *E. coli* DH5α, plated in triplicate on McConkey or LB agar plates, and incubated for 24 h at 37°C. Data shows the CFU ratio of *E. coli* (used as prey bacteria) to *S. arizonae* (used as attacker bacteria), normalized to the inoculum ratio and expressed as log_10_. Error bars indicate standard error. Statistical significance was determined using a one-way ANOVA test followed by Tukey’s multiple comparisons test (* *p* < 0.05; **** *p* < 0.0001; ns, not significant).

In the present study, we evaluated the contribution of T6SS_SPI-20_ and T6SS_SPI-21_ to interbacterial competition by *S. arizonae* serovar IIIa 62:z4,z23:- strain RSK2980 (hereafter referred to as *S. arizonae* RSK2980) and performed an *in silico* analysis of SPI-20 and SPI-21 T6SS gene clusters to identify putative effector and cognate immunity proteins. Our results show that *S. arizonae* RSK2980 outcompeted a susceptible *E. coli* strain in the presence of bile salts, as reported in the case of *S*. Typhimurium (Sana et al., 2016) and *S*. Dublin (Schroll et al., 2019). In addition, we established that both T6SS_SPI-20_ and T6SS_SPI-21_ contribute to interbacterial competition by this strain. Subsequently, a comprehensive bioinformatic analysis identified a novel potential E/I module encoded within SPI-21 (*SARI_02625*/*SARI_02624*), in addition to the previously predicted VgrG2b and VgrG-NucSe1 homologs encoded within SPI-20 and SPI-21, respectively (Figure 1A). Sequence and structure-based analyses of SARI_02625 revealed 3 putative functional domains, including a C-terminal region with high similarity to the LysF1 endolysin from *Escherichia coli* O157:H7 phage FAHEc1, suggesting peptidoglycan hydrolase activity. Finally, functional characterization by means of heterologous expression assays confirmed that the new candidate effector, along with the previously identified effectors, possesses antibacterial activity that can be neutralized by their cognate immunity proteins.

## 2 Results

### 2.1 T6SS_SPI-20_ and T6SS_SPI-21_ contribute to interbacterial competition in *S. arizonae*

Bile salts activate the expression of T6SS gene clusters of several bacterial pathogens (Crawford and Gunn, 2014; Sistrunk et al., 2016), including those encoded within SPI-6 and SPI-19 in *S*. Typhimurium and *S.* Dublin in which McConkey plate are used to induce T6SS-expression during bacterial growth (Brunet et al., 2015a; Sana et al., 2016; Schroll et al., 2019; Amaya et al., 2022) Therefore, we performed competition assays in either LB or McConkey agar using *E. coli* DH5α as the prey strain. To avoid cross-complementation among components of both T6SS gene clusters, as documented in other systems (Santos-Moreno et al., 2020), we generated mutant strains lacking the complete T6SS gene clusters of SPI-20 and/or SPI-21 in *S. arizonae* RSK2980. As shown in Figure 1B, the *E. coli* prey strain was significantly outcompeted by the *S. arizonae* wild-type strain only when co-incubated in McConkey agar, suggesting that bile salts promote the T6SS-dependent antibacterial activity of *S. arizonae*. Notably, strains lacking either T6SS_SPI-20_ or T6SS_SPI-21_ still outcompeted the prey strain at levels similar to those of the wild-type strain. Nonetheless*, S. arizonae* lacking both T6SSs was unable to outcompete the prey strain. These findings suggest that T6SS_SPI-20_ and T6SS_SPI-21_ confer a competitive advantage to *S. arizonae* RSK2980 over *E. coli* DH5α, and that the presence of bile salts activates both T6SSs. Of note, having both systems do not confer an additive advantage over having only one, suggesting functional redundancy between them.

### 2.2 SPI-20 encodes a putative zinc-dependent metallopeptidase with antibacterial activity

To identify novel T6SS effector proteins and their cognate immunity proteins, we performed a systematic analysis of each ORF encoded within the SPI-20 T6SS gene cluster of *S. arizonae* RSK2980. Firstly, we used the Bastion6 prediction pipeline (Wang et al., 2018a), a bioinformatics tool that employs amino acid sequence profiles, evolutionary information, and physicochemical properties to predict T6SS effectors. Secondly, to identify conserved domains and motifs associated with known T6SS effectors, the PROSITE, NCBI-CDD, Motif-Finder, and Pfam databases were used. In addition, functional predictions were made via HMM homology searches using the HHpred HMM-HMM prediction pipeline (Zimmermann et al., 2017) and through structure-based searches using Foldseek based on the predicted structure of each protein obtained with AlphaFold3 (Abramson et al., 2024). To identify if the predicted candidates could be part of an E/I module, we also performed a screen for putative immunity proteins by detecting signal peptides (SignalP), transmembrane domains (TMHMM), and bicistronic operons (Operon-mapper), as outlined by Taboada et al., (Taboada et al., 2018). In addition, we analyzed the SPI-20 T6SS gene cluster to identify potential unannotated ORFs that could encode putative effectors and cognate immunity proteins.

**Figure 2:**
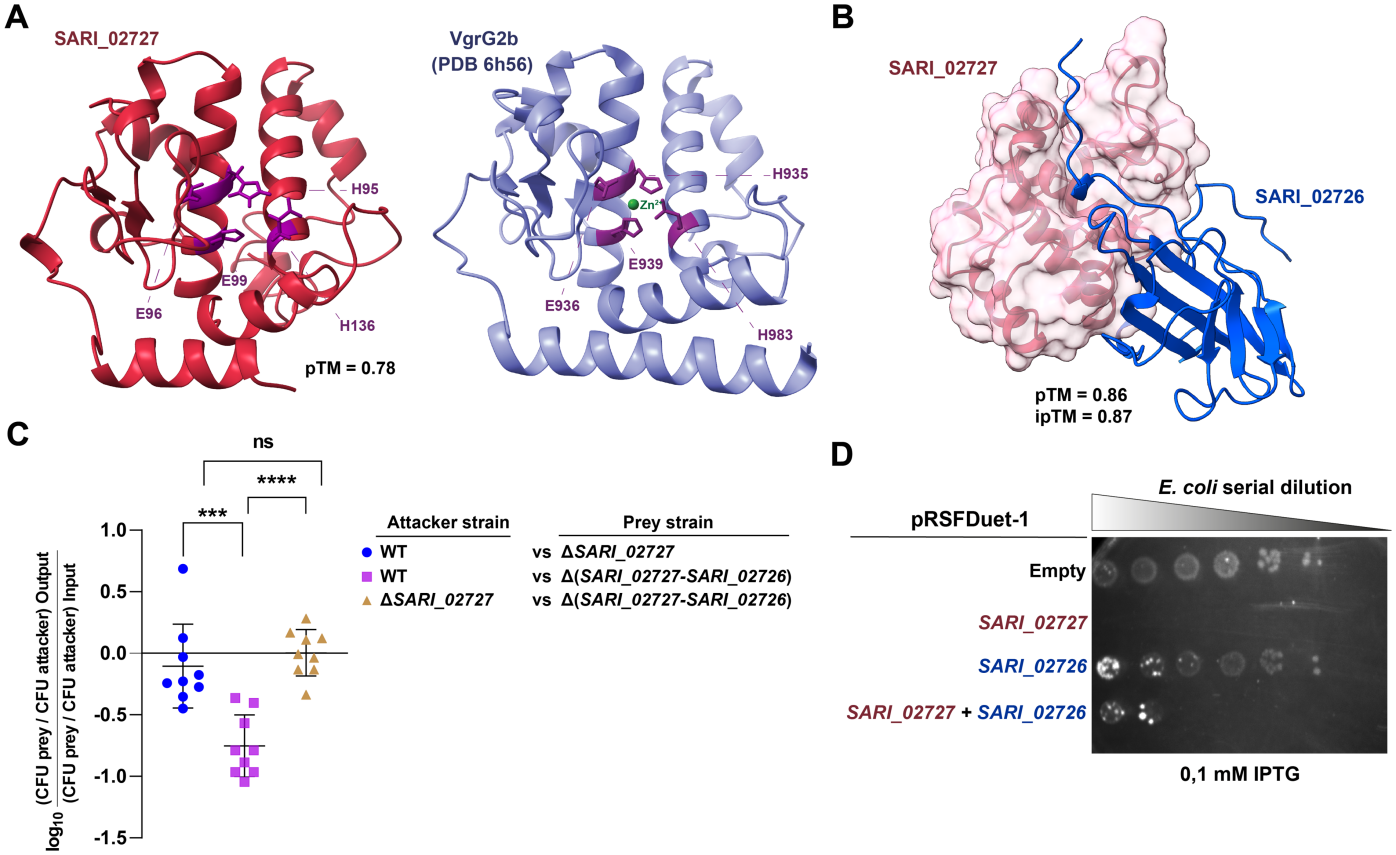
SARI_02727 contributes to antibacterial activity in *S. arizonae* RSK2980. **(A)** AlphaFold3 model of the predicted structure of SARI_02727 and PDB structure of VgrG2b of *P. aeruginosa* PAO1 (PDB 6 h56). The catalytic triads (HEXXH+E) of both proteins are highlighted in purple. **(B)** AlphaFold3 predicted protein–protein complex structure of SARI_02727/SARI_02726 T6SS E/I pair are shown with its corresponding ipTM score. **(C)** Interbacterial competition assay. Wild type and mutant strains Δ*SARI_02727* and Δ(*SARI_02727-SARI_02726*) were mixed at a ratio of 1:1 (attacker/prey) with *E. coli* DH5α, plated in triplicate on McConkey agar plates and incubated for 24 h at 37°C. Data shows the CFU ratio of *E. coli* (used as prey bacteria) to *S. arizonae* (used as attacker bacteria), normalized to the inoculum ratio and expressed as log_10_. Error bars indicate standard error. Statistical significance was determined using a one-way ANOVA test followed by Tukey’s multiple comparisons test (***p < 0.001; ****p < 0.0001; ns, not significant). **(D)** Heterologous expression assays. Survival of *E. coli* BL21(DE3) harboring pRSFDuet-1 plasmid expressing either *SARI_02727*, *SARI_02726* or both *SARI_02727/SARI_02726* genes were serially diluted in LB broth (1:4) and spotted onto LB agar plates containing Kan plus 0.1 mM IPTG to induce the synthesis of the effector, the immunity protein or both, respectively. The plates were incubated at 37°C for 24 h. Empty pRSFDuet-1 was used as control. SARI_02727 was targeted to the periplasm by the PelB signal peptide cloned in frame in the pRSFDuet-1 plasmid.

Our analysis confirmed the prediction of a previously identified putative T6SS effector protein and did not identify novel predicted effectors within SPI-20 (Table 1). This putative effector corresponds to SARI_02727 (Figure 2A), a protein previously identified as a putative homolog of the VgrG2b effector protein from *P. aeruginosa* (Wood et al., 2019). *SARI_02727* encodes an 189 amino acid protein with a modest Bastion6 score (0.611) predicted to be part of a bicistronic unit along with *SARI_02726.* Structure-based analysis of the AlphaFold3-predicted structure for SARI_02727 confirmed the structural similarity between SARI_02727 and the known structure of the VgrG2b effector protein. As shown in Figure 2A, SARI_02727 displays a high degree of similarity with the catalytic domain of VgrG2b, including the conserved HEXXH motif found in the catalytic site of zinc-dependent metallopeptidases (Amaya et al., 2024). The predicted catalytic amino acid side chains (H95, E96 and H136) in SARI_02727 coordinated with a zinc ion at the center (Figure 2A).

**Table 1:** Predicted T6SS effector and cognate immunity protein encoded in SPI-20 and SPI-21 of *S. enterica arizonae* RSK2980.

**ORF**	**T6SS gene cluster**	**Prediction**	**Size (aa)**	**Predicted Regions/Domains (database, score and region in amino acids)**
SARI_02727	SPI-20	Effector protein (Bastion6 score 0.611)	189	Motif-Finder: Disordered region (1-22aa)
				BLASTp: Type VI Rhs protein WP_155061119.1 (score 2E-118, identity 95.24%)
				AF3/Foldseek: Similarity to VrgG2b *P. aureginosa* (PDB 6H56) (score 2.64E-11)
SARI_02726	SPI-20	Immunity protein of SARI_02727	154	Phobius: Signal peptide (1-12aa)
				Pfam: PF24295 DUF7480 (score 8.9E-13, 36-128aa)
				NCBIfam: Putative T6SS immunity lipoprotein IPR054657 (score 2.5E-22, 15-127aa)
SARI_02603	SPI-21	Effector protein (Bastion6 score 0.611)	1196	NCBIfam: T6SS VgrG protein IPR006533 (score 9.5E-95, 26-500aa)
				Gene3D: Colicin/pyocin DNAse IPR037146 (score 2.7E-47, 1060-1196aa)
SARI_02602	SPI-21	Immunity protein of SARI_02603	98	CDD: Colicin/pyocin immunity protein IPR000290 (score 8.41E-27, 5-93aa)
SARI_02625	SPI-21	Effector protein (Bastion6 score 0.95)	1032	CDD: Endolysin/autolysin IPR033907 (score 1.469E-40, 882-1024aa)
				CDD: M23 peptidase (score 3.18E-6, 719-795aa)
				Prosite: EF-hand calcium-binding (555-588aa)
SARI_02624	SPI-21	Immunity protein of SARI_02625	276	Phobious: Signal Peptide (1-21aa)
				TMHMM: Transmembrane region (5-27aa)

As mentioned, *SARI_02727* is predicted to be part of a bicistronic unit with *SARI_02726*, suggesting that SARI_02726 is the immunity protein of the candidate effector protein. *SARI_02726* encodes a 154 amino acid protein with a periplasmic lipoprotein-type signal peptide (Sec/SPII). A sequence analysis revealed that SARI_02726 harbors the DUF7480 domain (IPR055903), not previously linked to T6SS function. We then used AlphaFold3 to model and predict if SARI_02727 and SARI_02726 could interact and constitute a T6SS E/I pair. As shown in Figure 2B, AlphaFold3 predicted an interaction between SARI_02727 and SARI_02726 with a high confidence score (ipTM: 0.87), suggesting that the two proteins could constitute a T6SS E/I pair.

To determine if SARI_02727 contributes to interbacterial competition in *S. arizonae*, we generated isogenic derivatives lacking either *SARI_02727* or the putative E/I module composed by *SARI_02727* and *SARI_02726*, and performed bacterial competition assays on McConkey agar. As shown in Figure 2C, the wild-type strain outcompeted the mutant lacking the putative E/I module, but not the ∆*SARI_02727* mutant, in agreement with the prediction that SARI_02726 corresponds to the immunity protein of SARI_02727. In addition, the ∆*SARI_02727* mutant was not able to outcompete the mutant lacking the putative E/I module.

Finally, to test the potential antibacterial activity of the predicted metallopeptidase domain of SARI_02727 and the neutralizing effect of the SARI_02726 immunity protein, we performed heterologous expression assays in *E. coli*. The fundamental premise underlying these assays is that the effector protein should cause a growth impairment when expressed in the appropriate localization in *E. coli*. This growth impairment should be counteracted, at least partially, by the concomitant production of the cognate immunity protein.

Thus, we transformed *E. coli* BL21(DE3) cells with derivatives of plasmid pRSFDuet-1 carrying genes encoding either the effector harboring an N-terminal PelB signal for periplasmic localization (pRSFDuet-1_SP_SARI_02727), the immunity protein (pRSFDuet-1_SARI_02726) or both (pRSFDuet-1_SP_SARI_02727/SARI_02726). As shown in Figure 2D, induction of SARI_02727 expression caused a drastic growth impairment in *E. coli* BL21(DE3). In addition, the co-expression of SARI_02727 and its cognate immunity protein (SARI_02726) partially restored cell growth (Figure 2D). At the same time, there was no apparent growth impairment when cells expressed only the immunity protein or carry the empty vector (Figure 2D). These results indicate that SARI_02727 is toxic to *E. coli* BL21(DE3) when targeted to the periplasm, and that SARI_02726 could partially neutralize its toxic effect, suggesting that they correspond to an E/I pair.

### 2.3 VgrG-NucSe1 (SARI_02603) contributes to the interbacterial activity of T6SS_SPI-21_ in *S. arizonae*

To date, only one effector protein, VgrG-NucSe1 (SARI_02603), has been predicted to be encoded within the SPI-21 T6SS gene cluster of *S. arizonae* (Blondel et al., 2009; Ho et al., 2017). VgrG-NucSe1 is an evolved VgrG protein harboring a C-terminal extension with homology to S-type pyocins (Blondel et al., 2009) (Table 1). Although it has been shown that the T6SS of *V. cholerae* can heterologously deliver the C-terminal region of VgrG-NucSe1 and possesses antibacterial activity (Ho et al., 2017), there is no direct report on the contribution of this effector to interbacterial competition in *S. arizonae*.

**Figure 3:**
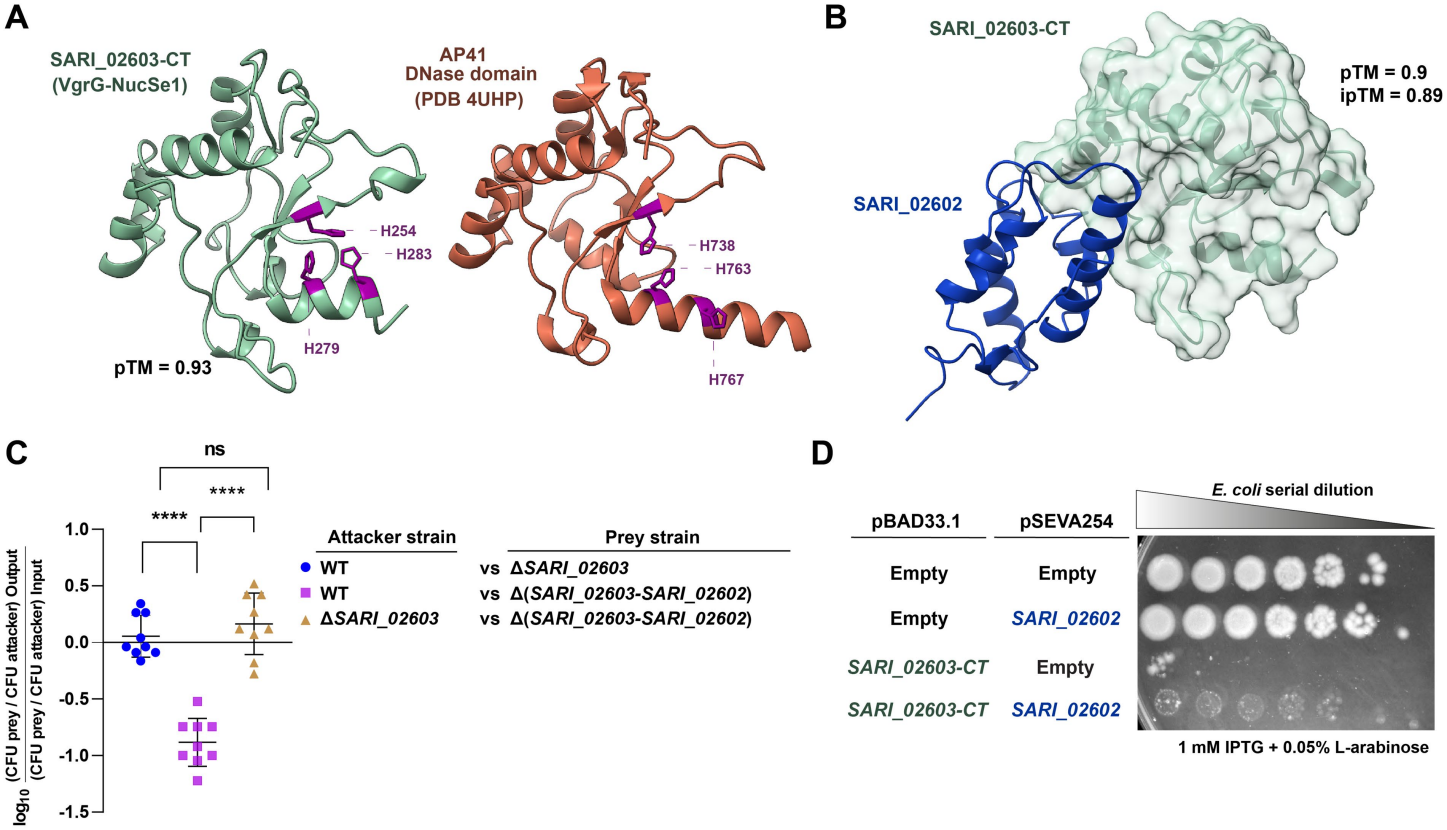
Contribution of the predicted C-terminal S-type pyocin domain of SARI_02603 to antibacterial activity in *S. arizonae* RSK2980. **(A)** AlphaFold3 model of the predicted structure of the C-terminal region of SARI_02603 and PDB structure of the DNase domain of pyocin AP41 (PDB 4UHP). The three catalytic histidines of both proteins are highlighted in purple. **(B)** AlphaFold3 predicted protein–protein complex structure of the SARI_02603/SARI_02602 T6SS E/I pair are shown with its corresponding ipTM score. **(C)** Interbacterial competition assay. Wild type and mutant strains Δ*SARI_02603* and Δ(*SARI_02603*-*SARI_02602*) were mixed at a ratio of 1:1 (attacker/prey) with *E. coli* DH5α, plated in triplicate on McConkey agar plates and incubated for 24 h at 37°C. Data shows the CFU ratio of *E. coli* (used as prey bacteria) to *S. arizonae* (used as attacker bacteria), normalized to the inoculum ratio and expressed as log_10_. Error bars indicate standard error. Statistical significance was determined using a one-way ANOVA test followed by Tukey’s multiple comparisons test (**** *p* < 0.0001; ns, not significant). **(D)** Heterologous expression assays. Survival of *E. coli* BL21(DE3) harboring pBAD33.1 plasmid expressing *SARI_02603-CT* and/or pSEVA254 plasmid expressing *SARI_02602* were serially diluted in LB broth (1:4) and spotted onto LB agar plates containing Cam and Kan plus 0.05% L-arabinose and/or 1 mM IPTG to induce the synthesis of the effector and the putative immunity protein, respectively. The plates were incubated at 37°C for 24 h. Empty pRSFDuet-1 and pSEVA254 were used as control.

First, we analyzed the predicted HNH nuclease domain within the C-terminal region of VgrG-NucSe1. A Foldseek analysis of the predicted structure generated by AlphaFold3 showed a significant similarity to the DNase domain of the AP41 pyocin from *P. aureginosa* (Figure 3A), including the three catalytic histidines of VgrG-NucSe1 (H254, H279, and H283 in *S. arizonae*). AlphaFold3 analysis also predicted with high confidence the interaction between the C-terminal region of VgrG-NucSe1 and its cognate immunity protein, SARI_02602 (Figure 3B).

To test if VgrG-NucSe1 contributes to the antibacterial activity of T6SS_SPI-21_ in *S. arizonae*, we performed interbacterial competition experiments between the wild-type strain and mutant strains lacking either VgrG-NucSe1 or both VgrG-NucSe1 and its cognate immunity protein SARI_02602. As shown in Figure 3C, the wild-type strain successfully outcompeted the mutant strain lacking both VgrG-NucSe1 and SARI_02602, but not the strain retaining the putative cognate immunity protein SARI_02602. In contrast, a mutant strain lacking VgrG-NucSe1 was unable to outcompete a mutant strain lacking the whole E/I module (Figure 3D). Finally, heterologous expression assays in *E. coli* strain BL21(DE3), showed that expression of the C-terminal region of VgrG-NucSe1 caused significant growth impairment, which was partially recovered by co-expression of the cognate immunity protein SARI_02602 (Figure 3D), in agreement with previous experimental data obtained in *V. cholerae* (Ho et al., 2017).

### 2.4 SPI-21 encodes a novel effector protein with predicted peptidoglycan hydrolase activity

To identify novel T6SS effector candidates within SPI-21, we performed a bioinformatic analysis similar to that described above for SPI-20. Our analysis identified one effector protein candidate encoded within SPI-21 (Table 1).

**Figure 4:**
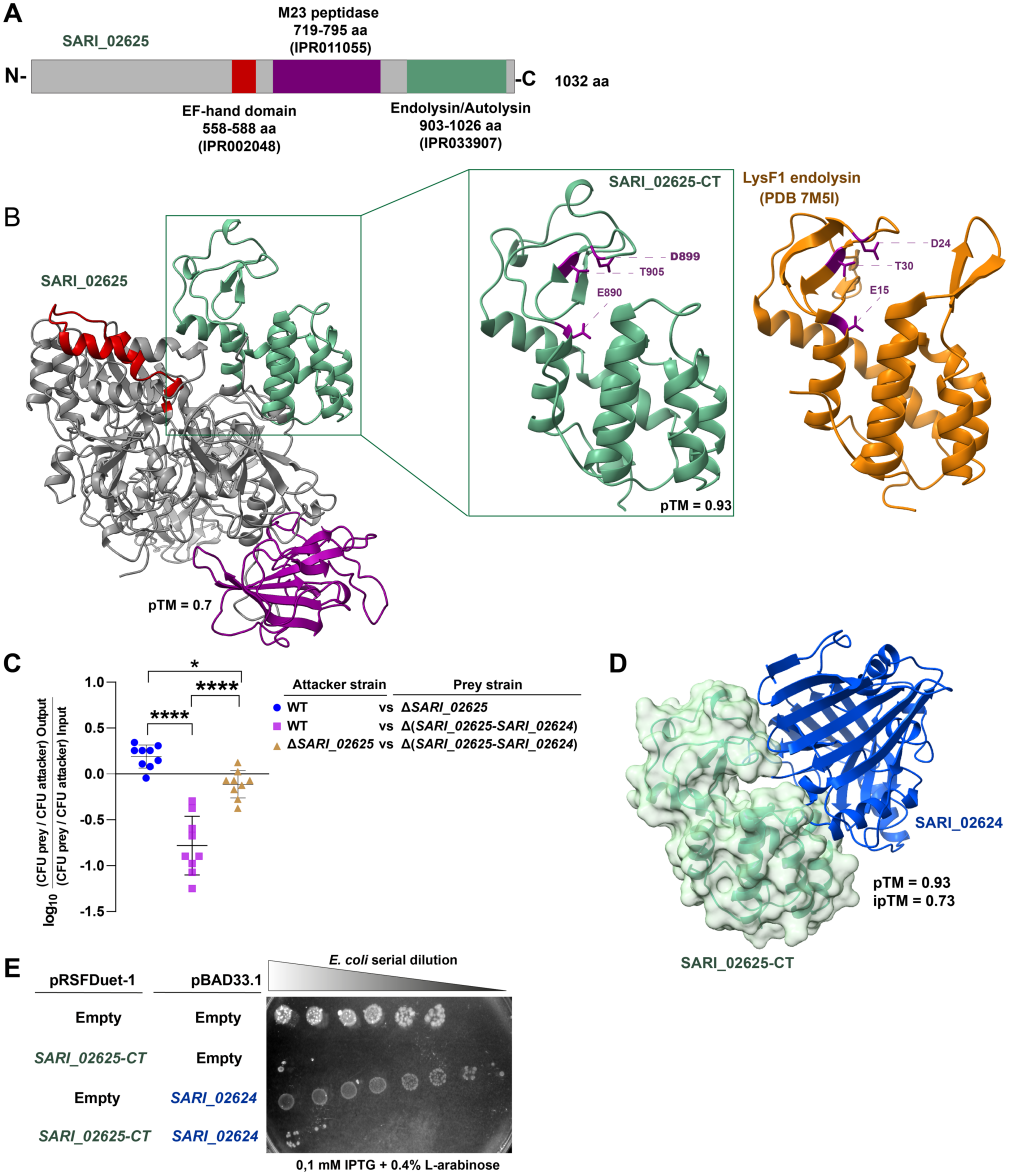
The SPI-21 T6SS gene cluster encodes a new putative antibacterial T6SS effector protein. **(A)** Scheme showing the main functional domains predicted within SARI_02625. **(B)** AlphaFold3 model of the predicted structure of SARI_02625 highlighting with different colors the predicted domains. A zoom of the C-terminal region of SARI_02625 is shown in the green box next to the PDB structure of the endolysin domain of the LysF1 endolysin from *E. coli* O157:H7 phage FAHEc1 (PDB 7M5I) for comparison. The catalytic triad ETD of both proteins is highlighted in purple. **(C)** Interbacterial competition assay. Wild type and mutant strains Δ*SARI_02625* and Δ(*SARI_02625*-*SARI_02624*) were mixed at a ratio of 1:1 (attacker/prey) with *E. coli* DH5α, plated in triplicate on McConkey agar plates and incubated for 24 h at 37°C and. Data shows the CFU ratio of *E. coli* (used as prey bacteria) to *S. arizonae* (used as attacker bacteria), normalized to the inoculum ratio and expressed as log_10_. Error bars indicate standard error. Statistical significance was determined using a one-way ANOVA test followed by Tukey’s multiple comparisons test (* *p* < 0.05; **** *p* < 0.0001; ns, not significant). **(D)** AlphaFold3 predicted protein–protein complex structure of SARI_02625/SARI_02624 T6SS E/I pair are shown with its corresponding ipTM score. **(E)** Heterologous expression assays. Survival of *E. coli* BL21(DE3) expressing pRSFDuet-1 plasmid expressing *SARI_02625-CT* or pBAD33.1 plasmid expressing *SARI_02624* were serially diluted in LB broth (1:4) and spotted onto LB agar plates containing Cam and Kan plus 0.1 mM IPTG and/or 0.4% l-arabinose to induce the synthesis of the effector and the putative immunity protein, respectively. The plates were incubated at 37°C for 24 h. Empty pRSFDuet-1 and pBAD33.1 were used as control.

This putative effector corresponds to SARI_02625 (Bastion6 score = 0.933), a 1,032 amino acid protein with three predicted functional domains in its C-terminal region (Figure 4A). These predicted domains correspond to an EF-hand domain (IPR002048), responsible for calcium-binding, a duplicated hybrid motif (IPR002048), frequently found in M23-related peptidases, and an endolysin/autolysin domain (IPR033907) found in peptidoglycan-degrading enzymes (Figures 4A,B). The presence of these three domains suggests that SARI_02625 is an antibacterial effector protein targeted to the bacterial periplasm (Figures 4A,B). In agreement with this, Foldseek analysis of the AlphaFold3-predicted structure of SARI_02625 revealed significant structural similarity of the endolysin/autolysin domain of SARI_02625 to the LysF1 endolysin of the *E. coli* O157:H7 phage FAHEc1 (Love et al., 2021), including the conserved E[DC]T catalytic triad (E890, D899, and T905 in SARI_02625) (Figure 4B). Our analysis also revealed that *SARI_02625* is part of a bicistronic unit along with *SARI_02624*. This ORF encodes a 276 amino acid protein with a periplasmic-targeting signal peptide (Sec/SPI), suggesting that SARI_02624 is the cognate immunity protein of the putative novel effector SARI_02625. Indeed, AlphaFold3 analysis predicted (although with moderate confidence) that SARI_02625 and SARI_02624 could interact, therefore constituting a T6SS E/I pair (Figure 4D).

To evaluate this, we performed interbacterial competition assays with the wild-type strain and mutant strains lacking either *SARI_02625* or both *SARI_02625* and *SARI_02624*. As shown in Figure 4C, the wild-type strain significantly outcompeted the mutant strain lacking the putative E/I pair, but not the strain lacking only the putative SARI_02625 effector protein. Furthermore, this mutant strain lacking the SARI_02625 effector protein could not outcompete the mutant strain lacking the putative E/I pair (Figure 4C). In addition, heterologous expression assays in *E. coli* showed that expression of the endolysin domain of SARI_02625 targeted to the periplasm, by adding the N-terminal region of PelB, caused significant growth impairment, which was partially recovered when the SARI_RS1220 protein was co-expressed (Figure 4E). Together, our results suggest that SARI_02625 and SARI_02624 conform a T6SS E/I pair encoded within SPI-21 in *S. arizonae.*

### 2.5 Distribution of the new candidate effectors identified in SPI-20 and SPI-21 in *Salmonella*

The characterization of new candidate T6SS effectors in *S. arizonae*, which harbor predicted antibacterial protein domains, prompted us to determine their distribution across *Salmonella*. To this end, the nucleotide sequence corresponding to the ORF encoding each candidate effector was used in tBLASTx searches in publicly available *Salmonella* genome sequences deposited in the NCBI database (August, 2025) and the distribution of each effector was determined. Our analysis revealed that SARI_02727, SARI_02603 and SARI_02625 candidate effectors encoded in the SPI-20 and SPI-21 T6SS gene clusters, respectively, are predominantly restricted to isolates of *S. enterica* subspecies *arizonae* and *diarizonae* (Supplementary Table S1).

## 3 Discussion

The T6SS is a versatile machine that delivers a wide range of effector proteins into prokaryotic and/or eukaryotic cells (Feria and Valvano, 2020; Hernandez et al., 2020). Therefore, it has evolved into a key molecular weapon in many bacterial pathogens. Five T6SS gene clusters have been identified in *Salmonella,* which are encoded within pathogenicity islands SPI-6, SPI-19, SPI-20, SPI-21, and SPI-22 (Blondel et al., 2009; Langridge et al., 2015). Although T6SS_SPI-6_ and T6SS_SPI-19_ contribute to interbacterial competition and host colonization in some serotypes, information on the contributions of T6SS_SPI-20_ and T6SS_SPI-21_ to these phenotypes in *S. arizonae* is lacking. Furthermore, there is also limited information regarding the repertoire of effector proteins encoded within these gene clusters. Nevertheless, the initial identification of an evolved VgrG protein with a C-terminal extension with homology to S-type pyocins encoded in the SPI-21 T6SS gene cluster of *S. arizonae* RSK2980 suggested that at least T6SS_SPI-21_ could be involved in antibacterial activity (Blondel et al., 2009). In this study, we report the first evidence of an antibacterial phenotype associated with T6SS_SPI-20_ and T6SS_SPI-21_ in *S. arizonae*, and present additional experimental data on three antibacterial effector proteins encoded within the SPI-20 and SPI-21 T6SS gene clusters.

Interbacterial competition assays revealed that *S. arizonae* strain RSK2980 exhibited bile-dependent antibacterial activity, which required the presence of both T6SS_SPI-20_ and T6SS_SPI-21_. Interestingly, bile salts are also responsible for inducing the activity of the T6SS_SPI-6_ and T6SS_SPI-19_ from *S*. Typhimurium and *S*. Dublin (Sana et al., 2016; Schroll et al., 2019; Amaya et al., 2022). The fact that phylogenetically distinct T6SSs respond to bile suggests a conserved regulatory mechanism governing T6SS genes expression and/or activity in *Salmonella*. It was recently shown that H-NS can function as c-di-GMP binding protein and that environmental and host-derived cues such as bile salts increase the intracellular levels of c-di-GMP, promoting the transcription of H-NS-repressed SPI-6 T6SS genes in *S*. Typhimurium (Brunet et al., 2015a; Li et al., 2023). Whether such regulatory mechanisms extend to *S. arizonae* is not currently known. Further work is needed to determine how MacConkey agar induces antibacterial activity by the T6SS_SPI-20_ and T6SS_SPI-21_ of *S. arizonae*.

The interbacterial competition assays also revealed apparent functional redundancy in antibacterial activity for both T6SS_SPI-20_ and T6SS_SPI-21_. This redundancy is highlighted by the fact that only when both systems were inactivated, the ability of *S. arizonae* to outcompete the *E. coli* prey was hampered. Since our experiments were performed using *E. coli* DH5α as the prey strain, this redundancy may also be prey-dependent, given that each T6SS gene cluster encodes a distinct set of effector proteins. This redundancy was not wholly unexpected, as it is similar to what we have reported previously for T6SS_SPI-6_ and T6SS_SPI-19_ in *S*. Dublin strain CT_02021853 (Amaya et al., 2022). The question of why *S. arizonae* has retained both T6SSs is still unanswered. Nevertheless, we cannot rule out that T6SS_SPI-20_ and T6SS_SPI-21_ may contribute to *Salmonella* fitness in other natural settings, such as the environment, or to colonization of reptiles, which are the natural host for *S. arizonae*. In these settings, both T6SS might not be redundant and each T6SS may target, or kill with different efficiencies, bacterial species within these environments. Further studies will be needed to address this issue.

Through bioinformatic analyses, interbacterial competition and heterologous expression assays, we identified and characterized three candidate T6SS effector proteins in *S. arizonae*. Within SPI-20, we characterized SARI_02727, a candidate effector previously predicted, but not experimentally validated, to belong to the VgrG2b family of zinc-metallopeptidase effectors previously identified in *P. aeruginosa* (Wood et al., 2019). Interbacterial competition and heterologous expression assays confirmed the antibacterial activity of the metallopeptidase domain of SARI_02727 and the protective effect of its cognate immunity protein, SARI_02726. Interestingly, in *P. aeruginosa*, VgrG2b is classified as a trans-kingdom effector protein, as it facilitates *P. aeruginosa* internalization into host cells by interacting with the host gamma-tubulin ring complex (Sana et al., 2015). Whether this could also occur with SARI_02727 remains an open question, as a trans-kingdom effector in *Salmonella* has not been identified yet. In addition, in *P. aeruginosa*, at least one other antibacterial effector protein can be loaded onto the VgrG2b spike (Berni et al., 2019), suggesting that other effector proteins could be loaded onto SARI_02727 as well.

Within SPI-21 we characterized the previously identified effector VgrG3-NucSe1 (Blondel et al., 2009; Wood et al., 2019). Interbacterial competition and heterologous expression assays confirmed the predicted antibacterial activity of VgrG3-NucSe1 and the protective effect of its cognate immunity protein SARI_02602, indicating that VgrG3-NucSe1/SARI_02602 is a T6SS E/I pair. In addition, we identified a novel effector protein encoded by *SARI_02625* within SPI-21. SARI_02625 harbors a predicted endolysin/autolysin domain (IPR033907) at its C-terminus. As in the case of SARI_02727, we demonstrate that a periplasmic version of the C-terminal region of SARI_02625 is responsible for the antibacterial activity of the SPI-21 T6SS gene cluster through heterologous expression assays, and that its toxicity is partially counteracted by its cognate immunity protein SARI_02624. Of note, SARI_02625 conserved the catalytic amino acid triad E[DC]T typically found in bacteriophage and bacterial lysozyme-like proteins that cleave the *β*(1,4)-glycosidic bonds between the N-acetylmuramic acid and the N-acetylglucosamine of the peptidoglycan (Love et al., 2021), and is the second peptidoglycan hydrolase candidate effector identified in the SPI-21 T6SS gene cluster of *S. arizonae* (Amaya et al., 2024). Indeed, the C-terminal domain of SARI_02625 has a high degree of structural homology compared to the LysF1 endolysin from *E. coli* O157:H7 phage FAHEc1 (Love et al., 2021), including the position of the predicted catalytic triad (E890, D899 and T906), strongly suggesting a peptidoglycan hydrolase activity. It is important to mention that even though we provide evidence of the contribution of these E/I modules to T6SS-dependent antibacterial activity of *S. arizonae*, further work is needed to provide evidence of T6SS-dependent secretion of these proteins.

Interestingly, the distribution analysis of SARI_02727, SARI_02603 and SARI_02625 T6SS candidate effectors in *Salmonella* genomes from the NCBI database revealed that they are predominantly restricted to *S. enterica* subspecies *arizonae* and *diarizonae* (Supplementary Table S1), suggesting a significant role in interbacterial competition and virulence for these subspecies. Altogether, our results indicate that both T6SS_SPI-20_ and T6SS_SPI-21_ contribute to interbacterial competition by *S. arizonae* RSK2980. In addition, this study broadens the repertoire and diversity of *S. arizonae* T6SS effector proteins. Nevertheless, the biochemical mechanisms underlying their antibacterial activity and their overall contribution to the environmental fitness and pathogenic potential of *S. arizonae* remain to be elucidated.

## 4 Materials and methods

### 4.1 Bacterial strains and growth conditions

The bacterial strains used in this study are listed in Table 2. Bacteria were routinely grown in Lysogeny Broth (LB) (10 g/L tryptone, 5 g/L yeast extract, 5 g/L NaCl) at 37°C with aeration. LB medium was supplemented with ampicillin (Amp; 100 μg/mL), kanamycin (Kan; 50 μg/mL), chloramphenicol (Cam; 20 μg/mL), or nalidixic acid (Nal; 15 μg/mL), as needed. LB medium was solidified by the addition of agar (15 g/L). For interbacterial competition assays, bacteria were grown on McConkey agar plates (BD) at 37°C for 24 h. For heterologous expression assays, bacteria were incubated at 37°C for 24 h on LB agar plates supplemented with Kan and/or Cam and the corresponding inducers (i.e., 0.1 mM IPTG and/or 0.4% L-arabinose).

**Table 2:** Bacterial strains and plasmids used in this study.

Strains	Features	Source or reference
*Escherichia coli*		
DH5α	F^−^ Φ80∆*lacZ*(M15) ∆(*lacZYA-argF*)*U169 deoR recA1 endA1 hsdR17*(r_k_^−^, m_k_^+^) *phoA supE44 thi-1 gyrA96 relA1 λ*^−^	Laboratory collection
BL21(DE3)	*E. coli B* F^−^ *ompT gal dcm lon hsdS_B_(r_B_^−^m_B_^−^) λ(DE3 [lacI lacUV5-T7p07 ind1 sam7 nin5]) [malB^+^]_K-12_(λ^S^)*	Laboratory collection
*Salmonella arizonae*		
RSK2980	Wild-type strain	Laboratory collection
ΔT6SS_SPI-20_	RSK2980 Δ(*SARI_02707*-*SARI_02736*)::Kan	This study
ΔT6SS_SPI-21_	RSK2980 Δ(*SARI_12055*-*SARI_12300*)::Cam	This study
ΔT6SS_SPI-20_ ΔT6SS_SPI-21_	RSK2980 Δ(*SARI_02707*-*SARI_02736*)::Kan Δ(*SARI_12055*-*SARI_12300*)::Cam	This study
Plasmids		
pKD46	*bla P_BAD_ bet gam exo oriR101*(TS), Amp^R^	Datsenko and Wanner (2000)
pCLF2	Red-swap redesigned vector, Amp^R^, Cam^R^	GenBank HM047089
pCLF4	Red-swap redesigned vector, Amp^R^, Kan^R^	GenBank EU629214
pRSFDuet-1	*E. coli* expression vector. Kanamycin resistance. IPTG-inducible recombinant protein expression vector. The vector contains two multiple cloning sites (MCS1 and MCS2), each of which is preceded by a T7 promoter/*lac* operator and a ribosome binding site. RSF replicon	Novagen
pBAD33.1	*E. coli* expression vector. Arabinose-inducible recombinant protein expression vector. Ampicillin resistance. P15A replicon	NovoPro
pET20b	IPTG-inducible recombinant protein expression vector. Ampicillin resistance. This vector adds an N-terminal PelB signal sequence for expression of proteins in the periplasm. ColE1 replicon	Novagen
pET20b_SP_2727	pET20b derivative. Expressing the full-length *SARI_02727* from *S. arizonae* RSK2980 with a PelB signal sequence at the N-terminal and a His6 tag at the C-terminal	This study
pET20b_SP_CT12255	pET20b derivative. Expressing the C-terminal domain of *SARI_02625* from *S. arizonae* RSK2980 with a PelB signal sequence at the N-terminal and a His6 tag at the C-terminal	This study
pRSFDuet-1_SP_2727	pRSFDuet-1 derivative. Expressing the full-length *SARI_02727* from *S. arizonae* RSK2980 with a PelB signal sequence at the N-terminal and a His6 tag at the C-terminal	This study
pRSFDuet-1_SP_2726	pRSFDuet-1 derivative. Expressing the full-length *SARI_02726* from *S. arizonae* RSK2980 with a His6 tag at the C-terminal	This study
pRSFDuet-1_SP_2727/2726	pRSFDuet-1 derivative. Expressing both the full-length *SARI_02727* from *S. arizonae* RSK2980 with a PelB signal sequence at the N-terminal and a His6 tag at the C-terminal, and the full-length *SARI_02726* with a a His6 tag at the C-terminal	This study
pRSFDuet-1_SP_CT12255	pRSFDuet-1 derivative. Expressing the C-terminal domain of *SARI_02625* from *S. arizonae* RSK2980 with a PelB signal sequence at the N-terminal and a His6 tag at the C-terminal	This study
pBAD33.1_12250	pBAD33.1 derivative. Expressing the full-length *SARI_02624* from *S. arizonae* RSK2980 with a His6 tag at the C-terminal	This study
pBAD33.1_Stype_pyocin_12160	pBAD33.1 derivative. Expressing the C-terminal domain of *SARI_02603* from *S. arizonae* RSK2980 with a His6 tag at the C-terminal	This study
pRSFDuet-1_Col_Imm_12155	pRSFDuet-1 derivative. Expressing the full-length *SARI_02602* from *S. arizonae* RSK2980 with a His6 tag at the C-terminal	This study

### 4.2 Standard DNA techniques

Plasmid DNA was isolated using the “QIAprep Spin Miniprep Kit” (QIAGEN, MD, USA). PCR products were purified using the “QIAquick PCR Purification Kit” (QIAGEN, MD, USA). Analysis of DNA samples was performed by electrophoresis in 1% agarose gels in 1X TAE buffer and visualized under UV light after RedGel (Biotium, CA, USA) staining. Primers were designed using the SnapGene software (www.snapgene.com) and are listed in Table 3. PCR reaction mixes contained 1X buffer, 2 mM MgCl_2_, 100 nM dNTPs, 100 nM of each primer, 100 ng of template DNA and 0.5 to 1 U of Phusion High-Fidelity DNA Polymerase (NEB, USA). Standard conditions for amplification were: 1 min at 98°C, followed by 30–35 cycles of 98°C for 10 s, 55°C to 72°C (according to the appropriate Tm for each primer pair) for 30 s and 72°C for a suitable time (15–30 s/kb), and a final extension step at 72°C for 10 min. All amplifications were conducted in a “MultiGeneTC9600-G” thermal cycler (LabNet, NJ, USA).

**Table 3:** Primers used in this study.

Primer	Sequence^a^
Mutagenesis	
SPI-20_H1 + P1	CGATCGTTTCTCCCTGGACGATATTTCGTTTTTCTCTGAG*GTGTAGGCTGGAGCTGCTTC*
SPI-20_H2 + P2	GTTCCTCCCGTAAATAATCAAATTCTCCTTTGCGCCATTC*CATATGAATATCCTCCTTAG*
SPI-20_OUT5	TCCGTTCCGCCACTATTTGA
SPI-20_OUT3	GGGGCGCGGGTATCGGTGCA
SPI-21_H1 + P1	GATTCGTTTGGGGGTAACCCACCGTTATATTCGTGCGGTC*GTGTAGGCTGGAGCTGCTTC*
SPI-21_H2 + P2	TAGCCGGTCTTTCTTTCACCTCACAGAGAGGCGCATTGCC*CATATGAATATCCTCCTTAG*
SPI-21_OUT5	AGGCACAAGGGGAGAAGGTG
SPI-21_OUT3	CAGTCTAAACTGAAGTAGCC
K1	CAGTCATAGCCGAATAGCCT
C3	CAGCTGAACGGTCTGGTTATAGG
Cloning	
pET20b_NcoI_SP_2727_F	CACACCATGGGTGGTTCTGGTATGACCATTCATGATGCCAG
pET20b_XhoI_SP_2727_R	CACACTCGAGTTAGTGGTGATGGTGATGATGAAACCCTCTTAGCGCCTCTT
pET20b_NcoI_SP_amid_F	CACACCATGGGTGGTTCTGGTGGGAAACAATTTATTAAGGA
pET20b_HindIII_SP_amid_R	CACAAAGCTTTTAGTGGTGATGGTGATGATGGTGAGATGCATCATATATAT
pET20b_OUT5_F	GGGAGACCACAACGGTTTCC
pET20b_OUT3_R	CCTTTCGGGCTTTGTTAGCA
pRSFDuet1_BamHI_2726ari_F	CACAGGATCCCATGGTGATAAAAAGATATAG
pRSFDuet1_HindIII_2726ari_R	CACAAAGCTTTTCAGAAACTTCAATTGAGC
pRSFDuet1_BamHI_ColImm_F	CACAGGATCCCGTGACTGAATTCAAAAAATC
pRSFDuet1_HindIII_ColImm_R	CACAAAGCTTTTAGTGGTGATGGTGATGATGTTTTTTTTCAGAGTCTTTGA
pBAD33.1_NdeI_12250_F	CACACATATGAAAACTTTACTTGTGTTTTT
pBAD33.1_HindIII_12250_R	CACAAAGCTTTTAGTGGTGATGGTGATGATGATATCCAGATTTAATAATAACAGC
pBAD33.1_NdeI_Stype_Pyo_F	CACACATATGCTGCGGCAGAAATCCCTCAC
pBAD33.1_SType_Pyo_HindIII_R	CACAAAGCTTTTAGTGGTGATGGTGATGATGCCTCCTATAGTGAATCTCAT
ACYCDuetUP1 Primer	GGATCTCGACGCTCTCCCT
DuetDown1 Primer	GATTATGCGGCCGTGTACAA
DuetUP2 Primer	TTGTACACGGCCGCATAATC
T7 Primer	GCTAGTTATTGCTCAGCGG
pBAD Forward	ATGCCATAGCATTTTTATCC
pBAD Reverse	GATTTAATCTGTATCAGG

a Italics indicate the region that anneals to the 5′ or 3′ end of the antibiotic resistance cassette used for the mutagenesis.

### 4.3 Construction of mutant strains

Derivatives of *S. arizonae* RSK2980 with deletions of the SPI-20 and/or SPI-21 T6SS gene clusters were constructed by the one-step inactivation procedure using the Lambda Red recombination system (Datsenko and Wanner, 2000), with modifications (Santiviago et al., 2009). The oligonucleotides used for mutagenesis (Table 3) were made with 40 bases at the 5′ ends identical to the ends of the corresponding deletion, and 20 bases at the 3′ ends that anneal with the 5′ or 3′ end of a Cam or Kan resistance cassette flanked by Flp recombinase target (FRT) sites present in plasmids pCLF2 (GenBank accession number HM047089) and pCLF4 (GenBank accession number EU629214.1), respectively. pCLF2 and pCLF4 were employed as templates for the corresponding amplification of PCR products. *S. arizonae* RSK2980 was transformed with plasmid pKD46, which allows the inducible expression of the *λ* Red recombination system in the presence of L-arabinose. Then, bacteria carrying pKD46 were grown to an OD_600nm_ of 0.6 at 30°C in LB broth supplemented with Amp and L-arabinose (10 mM). In the next step, bacteria were made electrocompetent through serial washes with ice-cold, sterile 15% glycerol, and transformed via electroporation with 500 to 600 ng of each PCR product. Transformants were selected at 37°C on LB agar supplemented with the corresponding antibiotic. The correct insertion of the corresponding antibiotic resistance cassettes in each mutant was confirmed by PCR amplification using suitable primers (Table 3). To avoid any potential off-target effect of the chromosomal insertion of PCR products, we used generalized transduction of the confirmed mutations to a *Salmonella* wild-type background by means of the transducing phage P22 HT105/1 *int-201*.

### 4.4 Interbacterial competition assays

Competition experiments to determine the ability of attacker strains to outcompete prey bacteria were conducted as described previously (Ma et al., 2018), with modifications. In brief, the attacker and prey bacteria were grown overnight in LB broth at 37°C. An aliquot (1 mL) of each culture was collected by centrifugation at 8,000 rpm for 2 min and the supernatant was discarded. Each bacterial pellet was washed three times in sterile PBS, adjusted to an OD_600nm_ of 0.5, and mixed at a 1:1 attacker-to-prey ratio. Then, aliquots (25 μL) of this mixture were spotted on McConkey agar plates in triplicate and incubated at 37°C for 24 h. This condition has been reported to induce expression of T6SS gene clusters in *Salmonella* (Schroll et al., 2019). After incubation, the bacterial spots were scraped from the McConkey agar plates, resuspended in 1 mL of sterile PBS, and colony-forming units (CFU) were determined by plating serial dilutions on LB agar supplemented with suitable antibiotics. CFU obtained from interbacterial competition experiments were used for data analysis to calculate a competition index (CI). The CI was calculated as the mean ratio of logarithmically converted CFU of the prey to attacker strains, normalized to the input ratio. Statistical significance was calculated using GraphPad Prism 9.0 software and a one-way ANOVA test followed by a Tukey’s multiple comparisons test.

### 4.5 Bioinformatic analyses

To identify putative T6SS effectors encoded within SPI-20 and SPI-21 T6SS gene clusters in *S. arizonae* RSK2980, each ORF of both pathogenicity islands was analyzed with the Bastion6 pipeline (Wang et al., 2018b) excluding those encoding the 13 structural components of the T6SS. ORFs exhibiting a Bastion6 score of at least 0.6 were designated as potential T6SS effectors. Each Bastion6 prediction was subjected to further analysis using tools implemented in the Operon-Mapper web server (Taboada et al., 2018) to determine its potential inclusion in a single transcriptional unit that also encoded a putative immunity protein. This term refers to a small protein with potential signal peptides (SignalP 6.0) and/or transmembrane domains (TMHMM 2.0). The identification of conserved functional domains and motifs in the candidate T6SS effectors was facilitated by the utilization of the PROSITE, NCBI-CDD, Motif-Finder, and Pfam databases (Kanehisa et al., 2002; Sigrist et al., 2013; Finn et al., 2014; Lu et al., 2019; Blum et al., 2020) which were integrated within the GenomeNet search engine. The E-value cutoff score was set at 0.01. Furthermore, for each effector and immunity protein identified, a biochemical functional prediction was conducted through HMM-based searches using the HHpred HMM-HMM comparison tool (Zimmermann et al., 2017). The analysis of predicted effector proteins is summarized in Table 1.

### 4.6 Plasmid construction for heterologous protein expression in *E. coli*

#### 4.6.1 *SARI_02727/SARI_02726* cloning strategy

For cloning the DNA sequence of putative SARI_02727 effector harboring an N-terminal PelB leader sequence and a C-terminal His-Tag; first, a PCR product containing the ORF of gene *SARI_02727* was generated using genomic DNA from *S. arizonae* RSK2980 and primers pET20b_NcoI_SP_2727_F and pET20b_XhoI_SP_2727_R (Table 3). The purified PCR product was digested with *Nco*I and *Xho*I and ligated to *Nco*I- and *Xho*I-digested vector pET20b, which encodes an N-terminal PelB leader sequence upstream of the multiple-cloning site, to generate plasmid pET20b_SP_2727 (Table 2). Then, a PCR product containing *SARI_02727* with the *pelB* leader sequence was generated using plasmid pET20b_SP_2727 as template and primers pET20b_OUT5_F and pET20b_OUT3_R (Table 3). The purified PCR product was digested with *Nde*I and *Xho*I and ligated into the multiple cloning site-2 (MCS2) of plasmid pRSFDuet-1 digested with the same enzymes to generate plasmid pRSFDuet-1_SP_2727 (Table 2). For construction of the plasmid used for heterologous expression of the immunity protein SARI_02726 fused to a C-terminal His-Tag in *E. coli*, the ORF of gene *SARI_02726* was amplified using genomic DNA from *S. arizonae* RSK2980 and primers pRSFDuet1_BamHI_2726ari_F and pRSFDuet1_HindIII_2726ari_R (Table 3). The amplification product was digested with *BamH*I and *Hind*III, and cloned into the MCS2 of plasmids pRSFDuet-1 and pRSFDuet-1_SP_2727 digested with the same enzymes to generate plasmids pRSFDuet-1_SP_2726 and pRSFDuet-1_SP_2727/2726, respectively (Table 2).

#### 4.6.2 *SARI_02625/SARI_02624* cloning strategy

For cloning the DNA sequence of the putative C-terminal toxic domain of SARI_02625 (residues 903 to 1,032) harboring an N-terminal PelB leader sequence and a C-terminal His-Tag, a PCR product containing the ORF of gene *SARI_02625* encoding the putative Lyz_endolysin_autolysin domain (SARI_02625-CT) was generated using genomic DNA from *S. arizonae* RSK2980 and primers pET20b_NcoI_SP_amid_F and pET20b_HindIII_SP_amid_R (Table 3). The purified PCR product was digested with *Nco*I and *Xho*I and ligated to *Nco*I- and *Xho*I-digested vector pET20b, which encodes a N-terminal PelB leader sequence upstream of the multiple-cloning site, to generate plasmid pET20b_SP_CT12255 (Table 2). Then, a PCR product containing the sequence encoding the C-terminal domain of *SARI_02625* with the *pelB* leader sequence was generated using plasmid pET20b_SP_CT12255 as template and primers pET20b_OUT5_F and pET20b_OUT3_R (Table 3). The purified PCR product was digested with *Nde*I and *Xho*I and ligated into the MCS2 of plasmid pRSFDuet-1 digested with the same enzymes to generate plasmid pRSFDuet-1_SP_CT12255 (Table 2). For construction of the plasmid used for heterologous expression of the immunity protein SARI_02624 fused to a C-terminal His-Tag in *E. coli*, the ORF of gene *SARI_02625* was amplified using genomic DNA from *S. arizonae* RSK2980 and primers pBAD33.1_NdeI_12250_F and pBAD33.1_HindIII_12250_R (Table 3). The amplification product was digested with *Nde*I and *Hind*III and cloned into pBAD33.1 digested with the same enzymes to generate plasmid pBAD33.1_12250 (Table 2).

#### 4.6.3 *C-terminal_SARI_02603/SARI_02602* cloning strategy

For cloning the DNA sequence encoding the predicted C-terminal S-Type pyocin domain of SARI_02603 (residues 903 to 1,032) harboring a C-terminal His-Tag, a PCR amplicon containing the DNA region of S-Type pyocin domain was made using genomic DNA and primers pBAD33.1_NdeI_Stype_Pyo_F and pBAD33.1_SType_Pyo_HindIII_R (Table 3). Then, the purified PCR product was digested with *Nde*I and *Hind*III and ligated to pBAD33.1 digested with the same restriction enzymes, to generate plasmid pBAD33.1_Stype_pyocin_12160 (Table 2). To clone the cognate immunity protein of SARI_02603, the PCR product containing the DNA sequence of *SARI_02602* and a C-terminal His-Tag was generated using primers pRSFDuet1_BamHI_ColImm_F and pRSFDuet1_HindIII_ColImm_R (Table 3). Then, the amplicon was digested with *BamH*I and *Hind*III and cloned into the multiple cloning site-1 (MCS1) of plasmid pRSFDuet-1 digested with the same enzymes to produce plasmid pRSFDuet-1_Col_Imm_12155 (Table 2).

### 4.7 Heterologous toxicity assays

The growth inhibition caused by SARI_02727 effector in *E. coli* BL21(DE3) was determined by growth on solid media in the presence or absence of the inducer IPTG. Overnight cultures of *E. coli* BL21(DE3) cells with pRSFDuet-1 plasmid containing *SARI_02727*, *SARI_02726* or both genes were diluted 4-fold and aliquots (5 μL) were spotted onto LB agar plates containing Kan plus 0.1 mM IPTG to induce the synthesis of either the effector, immunity protein or both proteins, respectively. The plates were incubated at 37°C for 24 h and the inhibition of bacterial growth was visually determined. *E. coli* BL21(DE3) containing empty pRSFDuet-1 was used as control.

To evaluate the ability of SARI_02625-CT to inhibit bacterial growth and the protection conferred by its cognate immunity protein (SARI_02624), overnight cultures of *E. coli* BL21(DE3) co-expressing the C-terminal domain of effector SARI_02625 from plasmid pRSFDuet-1_SP_CT12255 and its immunity protein SARI_02624 from plasmid pBAD33.1_12250 were serially diluted in LB broth (1:4) and aliquots (5 μL) were spotted onto LB agar plates containing Kan and Cam, plus 0.1 mM IPTG and/or 0.4% L-arabinose to induce the synthesis of the effector and immunity protein, respectively. The plates were incubated at 37°C for 24 h and bacterial growth inhibition was determined. Derivative strains of *E. coli* BL21(DE3) containing either pRSFDuet-1 and pBAD33.1 empty vectors; pRSFDuet-1_SP_CT12255/pBAD33.1 empty and pRSFDuet-1 empty/ pBAD33.1_12250 were used as controls.

To evaluate the bacterial toxic activity of the S-Type pyocin domain of SAR_02603 and its neutralization by its cognate immunity protein SARI_02602, a similar approach as described above for SARI_02625-CT/SARI_02624 was conducted. In this case, the heterologous expression assay was performed on LB agar plates containing Kan and Cam, plus 0.05% L-arabinose and/or 1 mM IPTG to induce the synthesis of the effector (pBAD33.1_Stype_pyocin_12160) and immunity protein (pRSFDuet-1_Col_Imm_12155), respectively.

### 4.8 Protein structure prediction and homology modelling

Protein structure models of SARI_02727, SARI_02625 and the C-terminal domain of SARI_02603 were obtained using I-TASSER (Yang et al., 2015), a protein structure homology-modeling server, and AlphaFold3 (Abramson et al., 2024) to predict effector-immunity protein interactions. The best model was used in downstream analyses (i.e., the rank_001 model). Protein structure visualization, template alignment, and superposition were performed using Pymol v3.1 and Mathmaker of UCSF ChimeraX (Pettersen et al., 2021). Protein structure searchers were performed with the Foldseek server (van Kempen et al., 2024).

### 4.9 Analysis of SPI-20 and SPI-21 T6SS effectors distribution

The DNA sequence encoding each T6SS effector identified in this study was subjected to tBLASTx analyses to find orthologs in all *Salmonella* genome sequences deposited in the NCBI database (August, 2025). The selection of positive matches was based on a 90% identity and 90% sequence coverage threshold. The conservation of sequences was determined by multiple sequence alignments using T-Coffee Expresso (Notredame et al., 2000), MAFFT (Katoh et al., 2017), and ESPript 3 (Robert and Gouet, 2014). Comparative genomic analyses of T6SS gene clusters were performed using Mauve (Darling et al., 2004) and EasyFig v2.2.5 (Sullivan et al., 2011). The analysis of nucleotide sequences was conducted using Artemis version 18 (Rutherford et al., 2000).

## Data Availability

The raw data supporting the conclusions of this article will be made available by the authors, without undue reservation.
